# A Multimodal Virtual Reality Data Acquisition Platform and Dataset to Assess Systemic Human Cognitive States

**DOI:** 10.1038/s41597-025-06384-9

**Published:** 2025-12-09

**Authors:** Ayca Aygun, Giles Blaney, Zachary Haga, Thomas McWilliams, Julia Mertens, J. P. de Ruiter, Nathan Ward, Matthias Scheutz

**Affiliations:** 1 Tufts University Department of Computer Science, Medford, MA 02155 US; 2 Tufts University Department of Biomedical Engineering, Medford, MA 02155 US; 3 Tufts University Department of Psychology, Medford, MA 02155 US

**Keywords:** Human behaviour, Biomedical engineering, Databases, Decision making

## Abstract

In recent years, there has been an increasing interest in human-machine teaming for search and rescue operations, deep space missions, and agricultural tasks, among others. To be effective teammates, artificial agents should be able to detect and be responsive to systemic human cognitive states such as workload, sense of urgency, mind wandering, interference, and distraction. Here, we introduce an experimental paradigm and a comprehensive multimodal dataset that provides the necessary data for analyzing the relationships among multiple systematic human cognitive states, enables the development of robust prediction models of these states, and details the framework for developing new experiments. The introduced experimental setup allows for the synchronized real-time recording of multiple data streams from various sensing devices including fNIRS, EEG, pupillometry, respiration, electrodermal activity, and plethysmography, and is applicable in many other interactive task settings where human performance needs to be monitored. The dataset was acquired from 80 subjects performing a driving task and several secondary tasks including car braking events, dialogue communications, and tactile stimulations.

## Background & Summary

There is a growing interest in developing artificial agents for mixed-initiative human-machine teams that are sensitive to human cognitive states and can adapt their behavior and interactions based on their moment-by-moment assessments of those states to better support their human teammates. For example, artificial agents could reduce their interaction frequency to reduce the cognitive load of stressed humans, or they could prompt humans with specific actions when they are distracted or mind-wandering to get them back on track. Overall, the expectation for these interventions is that artificial agents, by being able to track human cognitive states and proactively selecting interventions, can contribute to increasing human and thus team performance. To study human cognitive states, in particular, systematic cognitive states such as workload, mind wandering, distraction, etc. and determine which sensory information might allow for the development of robust computational models to detect them, a task-general multi-modal experimental data recording paradigm is needed that allows for the collection of a comprehensive dataset consisting of any potential sensory modality that could contribute to the development of computational models.

Here, we present such an experimental setting with a comprehensive data collection framework that can be directly applied to different tasks and performance environments. We also present an open-access multi-modal multitasking dataset created from extensive experiments in a simulated driving task using the proposed recording and data collection paradigm. The seated driving setting allowed us to mount a suite of different physiological sensors on subjects that based on the literature were hypothesized to allow for the inference of different systemic human cognitive states present at different times during the driving task, including states of workload, sense of urgency, mind wandering, cognitive interference, and distraction. The dataset, which is larger and more comprehensive than comparable driving datasets, includes both physiological data such as EEG, fNIRS, pupillometry, respiration, electrodermal activity, and beat-to-beat plethysmography, as well as behavioral data such as various events occurring during driving from eighty participants, each performing two driving sessions while also performing different secondary tasks such car braking happenings, dialogue communications and the responding to tactile stimulations in the DRT task (^1^).

There are several publicly available datasets that examine various cognitive state estimations within driving simulation environments (e.g., see^2–4^ for comparable research studies). For example,^5^ developed a real-world driving dataset using ECG, skin conductance, and body temperature to study drivers’ workload levels. However, the dataset only contains ten participants which both limits the generalizability of the findings to larger populations and the applications of data analysis and machine learning techniques. In contrast,^6^ introduced a comprehensive dataset to examine drivers’ physiological and behavioral states such as drowsiness, mental workload, and situation awareness during conditionally automated driving. This dataset contains data from 346 drivers acquired while the participants were performing six experiments. While the dataset includes different physiological signal modalities such as ECG, EDA, and respiration, it is lacking several other relevant physiological signal modalities, notably EEG, fNIRS, and pupillometry, which possess considerable potential for evaluating individuals’ systemic cognitive states. To the best of our knowledge, the present dataset is the most comprehensive in terms of simultaneously recorded physiological signals and thus provides a wealth of opportunities for future data analyses, model and theory developments, machine learning applications, and novel task environments to enable comparison data using the same experimental data collection framework.

Our goal in publishing this exclusive dataset and proposing the experimental data collection framework was to expedite the research in the detection and the tracking of systemic human cognitive states with applications in human-machine interaction and teaming, including but not limited to the following:

- Exploring systemic human cognitive states and their effects on the overall team performance in multi-modal multitasking human-machine interactive systems.
- Evaluating the utility of multiple physiological biomarkers acquired from different signal modalities, including EEG, pupillometry, arterial blood pressure, fNIRS, skin conductance, and respiration, alone or combined, for assessing systemic human cognitive states.
- Validating the efficiency of state-of-the-art data analysis and machine learning algorithms for predicting different systemic human cognitive states such as cognitive workload, sense of urgency, mind wandering, cognitive interference, and distraction.
- Studying the dynamics of the relationship between different pairs of human cognitive states, such as examining the association between mind wandering and cognitive workload or investigating how the overall workload changes as a result of consecutive sense of urgency occurrences.

In addition, the data recording framework which allows for the time-stamped synchronized collection of different real-time data streams across a diverse set of devices will hopefully encourage comprehensive data recordings in other tasks and performance environments. We thus believe that the dataset and experimental framework will be of great utility to human factors psychologists, cognitive and computer scientists, computational cognitive modelers, human-computer interaction researchers, and human factor engineers alike in their effort to better understand the impact of human systematic cognitive states on human performance and ways to modulate them to achieve better human performance.

It is important to note that while the dataset was previously used to estimate overall workload based on EEG, fNIRS, and eye gaze data^7–9^, it was not previously made public, nor did the previous pulications provide a comprehensive documentation or tools necessary for broader reuse. In contrast, this paper provides a complete description of the dataset that enables other researchers to fully leverage it for new analyses and new methodological developments. Specifically, we provide extensive technical documentation covering the experimental protocol, sensor design and placement, synchronization of multiple signal streams, software architecture, and task structure. Additionally, all custom code used for data acquisition, preprocessing, and initial formatting is made publicly available, allowing full transparency and reproducibility.

The dataset also supports extensions to multimodal fusion models, real-time inference of cognitive states, and adaptive human-machine interaction systems. Furthermore, the dataset includes rich behavioral and physiological markers associated some other cognitive states including distraction and interference that were not examined in our earlier work. This opens the door to new investigations into attentional lapses, error prediction, decision-making under uncertainty, and the impact of cognitive state change in complex tasks.

The longitudinal nature of the dataset, with 80 participants completing two experimental sessions each, provides a valuable structure for within-subject and across-session analyses. This enables research into learning effects, fatigue, individual variability in cognitive dynamics, and cross-session model generalization.

Beyond these applications, the dataset also lends itself to the development and validation of advanced signal processing techniques, including artifact removal, noise reduction, and signal enhancement in multimodal data streams. Researchers focused on algorithmic development for temporal alignment, multimodal synchronization, and signal feature extraction can benefit from the dataset’s complexity and richness. Additionally, the multimodal nature of the data supports exploratory analyses using deep learning architectures such as LSTMs, transformers, or self-supervised contrastive learning approaches for cognitive state representation.

In sum, while the dataset has been previously employed in a focused study, this paper is essential for making it an accessible, usable, and extensible resource for the research community. By offering full access, technical transparency, and proposals for novel analytic approaches, this dataset aligns with open science principles and provides significant added value for a wide range of cognitive and computational research domains.

## Methods

### Participants

One hundred thirteen people, who were recruited from the local community, participated in the study during 2019 and 2020 at Tufts University. A total of 31 participants were excluded from the final analysis for different reasons: 14 participants were excluded because of technical issues, and 19 participants withdrew from the study before study completion due to discomfort and simulator sickness. The final dataset consisted of 80 participants with the mean and the standard deviation of the age of 19.9 yr and 3.31 yr, respectively. 46.8% of the participants self-identified as female, and the remainder as male. All recruited participants were right-handed, had normal or corrected to normal vision, had a valid driver’s license, and drove at least one day a week on average. Participants received either a payment of $20 (n = 18) or two hours of research credits for an introductory Psychology course (n = 62).

The dataset, which contains 80 participants each completing two separate experimental sessions, offers a rich foundation for both within-subject and across-session analyses. This structure supports investigations into temporal dynamics of cognitive states, including learning effects, cognitive fatigue, and the stability of individual differences over time. It also enables the evaluation of model generalizability across sessions and the development of personalized cognitive models that adapt to changes in user state or performance trajectories.

### Apparatus & Instrumentation

#### Driving Simulator

**Fig. 1:**
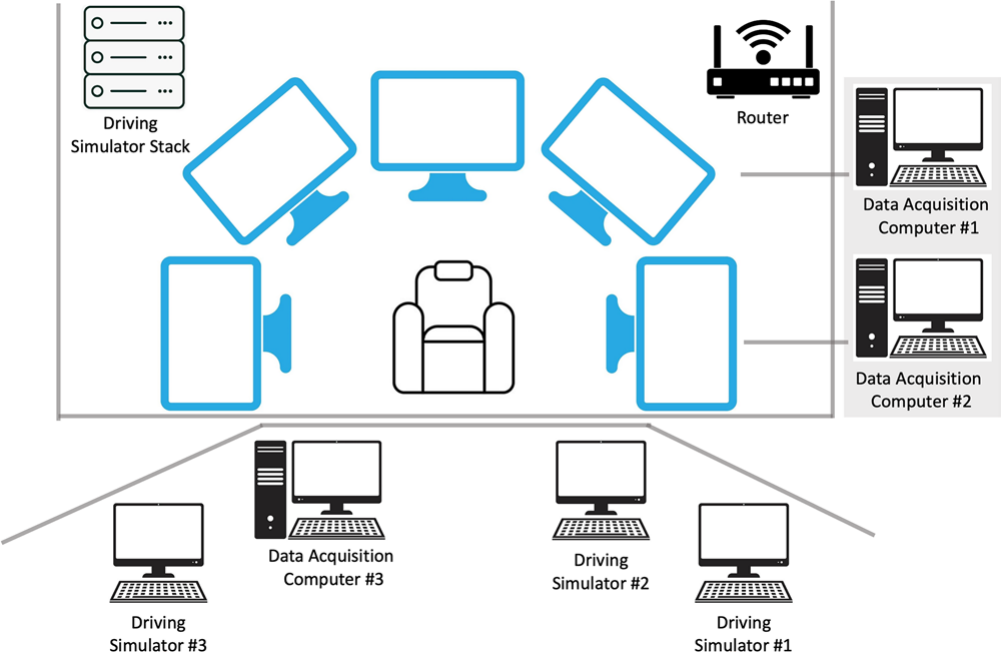
Schematic of the physical layout of the experimental environment.

We used a medium fidelity partial-cab driving simulator that had a working steering wheel, brake pedal, accelerator pedal, and Park, Reverse, Neutral, Drive, and Low (PRNDL) gear shift. The dashboard, the driver’s car seat, and the console were taken from a 2010-era Ford Fusion. The peripheral audiovisual equipment (AV) cart with cushions was placed on the left of the participant to provide the participant with an armrest where the driver door’s armrest would be in the actual automobile. The simulator used five 45 in LCD monitors to display the forward road scene with a 180 deg field of view. The simulated but functioning side mirrors were located adjacent to the main video at approximately their realistic locations. Figure 1 shows the schematic of the physical layout of the simulation.

The proposed driving simulation included a straight four-lane highway (two lanes in each direction). The highway environment was lined with trees, had clear weather, and took place during the day. The traffic was light with cars in the left lane passing the driver roughly every 30 s. The posted speed limit was 65 mph. The simulator captured images, generated driving data, and recorded them at 60 Hz. Audio of the driving environment was presented to participants through noise-canceling earbuds (Bose QuietComfort 20).

**Fig. 2:**
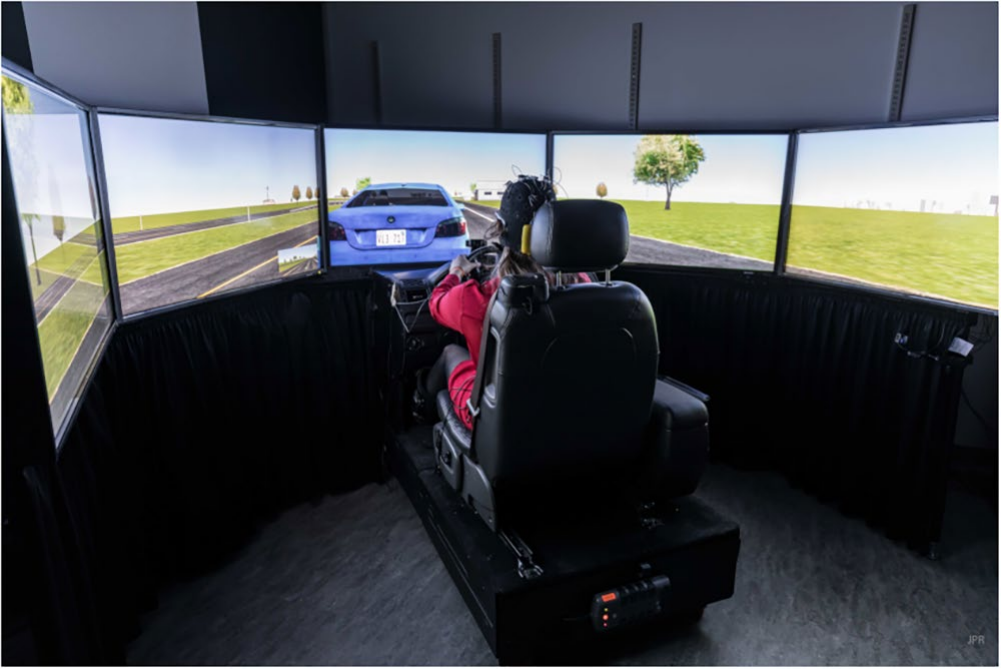
The simulator showing the moment before a braking event when another car had just cut in front of the driver (the person in the image is a member of the study team and has given consent for the image to be published).

Software and hardware for the driving simulator were provided by the Research Triangle Institute (RTI) (Ann Arbor, MI). RTI wrote custom software to send out the simulator’s experimental time to a LSL network socket. The LSL protocol recorded that datum along with the synchronized networked time stamp (see the next section). All other simulator data was written to a file in an array with the simulator’s experimental time at which the other simulator data was recorded. An example participant is depicted in Fig. 2 illustrating (a) the front camera, (b) the side camera, and (c) the world camera.

#### LSL

The data acquisition system was built using an isolated, dedicated high-speed router (ASUS, GT-AC5300) and LSL software libraries either hand-written or supplied by a specific piece of equipment’s manufacturer. LSL is an open-source software library developed to facilitate multi-modal data stream synchronization by attaching a universal network timestamp and by creating a data socket that other networked computers can subscribe to receive. LSL periodically checks clock offsets to correct for nonlinearities across networked data acquisition devices, thus can synchronize distributed data collection to a universal timestamp. This system allowed us to design a robust data acquisition platform to acquire different signal modalities, such as eye gaze and EEG, and synchronize them to the exact event timestamps, which is essential for an accurate analysis.

#### fNIRS

**Fig. 3:**
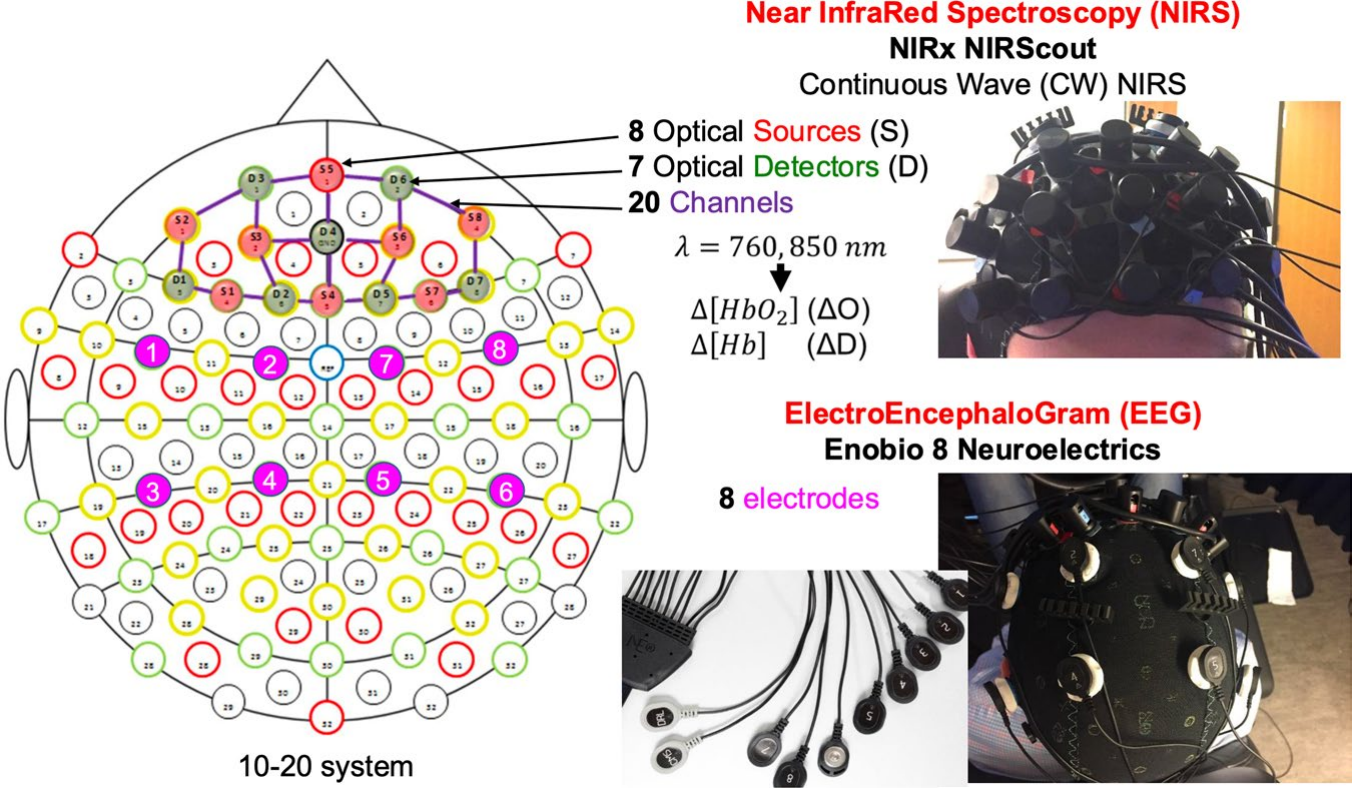
Site locations for the pre-frontal fNIRS optode montage and EEG electrode placements.

fNIRS was measured by a NIRScout (NIRx Medical Technology, Berlin, Germany) device, which consisted of “light emitting diode” (LED) source pairs (at wavelengths of 760 nm and 850 nm) fiber bundle coupled “photo-diode” (PD) detectors. These optical data were collected at 7.81 Hz and recorded with the LSL protocol from an integrated LSL network socket available in the manufacturer’s software package, NIRStar. Optical data was acquired from a pre-frontal optode configuration using NIRx’s head caps, which were marked with the standard international 10-10 site positions seen in Fig. 3. The optical signal calibration and signal integrity were conducted and verified with the NIRStar software package during the last stage of the participants’ preparation.

In the simulation scenario introduced in this paper, we included the fNIRS data (along with the other systemic physiological signal modalities including heart rate, arterial blood pressure, respiration rate, skin conductance, etc) as a complementary modality that can provide partial information about cerebral hemodynamics, even in the presence of contamination. Importantly, the fNIRS data are not intended to be analyzed in isolation as a pure measure of cortical activity. Rather, in the context of this multimodal dataset, they serve as one source of information that may contribute meaningfully to joint analyses, particularly when combined with other physiological signals that help characterize systemic states. We encourage future users of the dataset to be mindful of these limitations and to interpret fNIRS signals with appropriate caution, especially in studies aiming to isolate neural activity.

#### EEG

EEG was collected at 500 Hz using an 8-channel Enobio (Neuroelectrics, Cambridge, MA, USA) system. 3.14 cm^2^ silver/silver chloride electrodes were placed at the international 10-10 system locations FC1, FC2, FC5, FC6, CP1, CP2, CP5, and CP6 (shown in Figure 3). The recording sites were grounded and referenced to the right ear lobe. The electrodes were mounted onto the NIRx’s fNIRS head cap, posterior to the optical sensors and detectors. The EEG signal quality was checked by using the manufacturer’s software package, Neuroelectrics Instrument Controller (NIC).

#### Eye tracking and pupillometry

A Pupil Core (Pupil Labs, Berlin, Germany) eye tracker was used to collect eye movements, pupil diameter, and blink rate. This eye tracker contained dual 200 Hz eye cameras and a 120 Hz world camera.

#### Physiological Monitoring Suite

Participants were also outfitted with several physiological monitoring systems. These systems were wired to an IPS100C Isolated power supply and an amplifier (BIOPAC Systems, Goleta, CA). The output from the IPS100C was digitized and passed to an auxiliary computer by a National Instruments USB-6001 data acquisition device. The physiological systems used in this experiment are listed as follows:

- A RSP100C respiration belt (BIOPAC Systems, Goleta, CA) was attached around the participant’s chest.
- On the participant’s left hand, skin conductance was measured with an EDA100C (BIOPAC Systems, Goleta, CA) electrodermal activity sensor.
- A NIBP100D (BIOPAC Systems, Goleta, CA) beat-to-beat finger plethysmography system was placed on the index and the middle finger of the participant’s left hand. A blood pressure cuff was attached to the participant’s left arm to calibrate the plethysmography signals.
- OXY100E (BIOPAC Systems, Goleta, CA) finger clip pulse oximeter was attached to the participant’s left thumb.
- Arterial blood pressure signals are acquired non-invasively using the CNSystems CNAP 500 monitor and further amplified and processed through the BIOPAC DA100C module for detailed physiological analysis.

This physiological monitoring suite of four data streams was collected at a 20 Hz sampling rate per stream.

#### Surveillance Cameras

A Logitech USB webcam was placed on top of the driving simulator’s forward monitor, and another was placed on the second monitor that was at approximately 45 deg angle to the seated participant during the study. The video was primarily used for monitoring the participant and the instrumentation and was also used to verify movement artifacts or experimental abnormalities (i.e., yawning or equipment dislodging). Custom software was written in C++ that recorded a “.avi” file at 10 fps to 15 fps. The video was recorded to a local hard drive, and the program created an LSL socket to document when a particular frame number was acquired. For ethical reasons, the video data are not available with the published dataset.

#### Audio Recordings

**Fig. 4:**
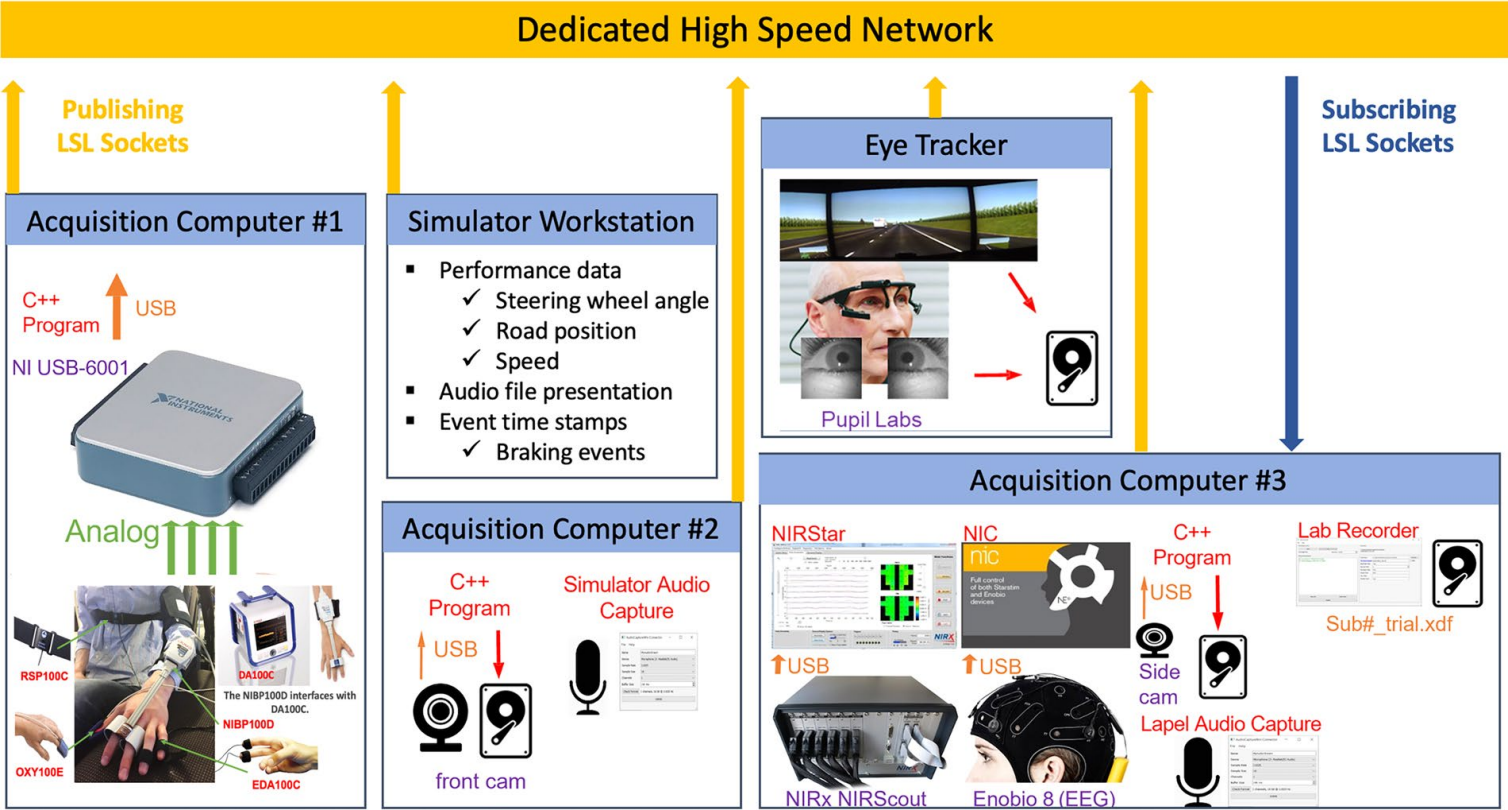
Network and software schematic of the data acquisition system.

We recorded two audio data streams: the simulator’s audio output and a lapel microphone attached to the front of the participant. Each data stream was recorded with LSL’s audio capture software (https://github.com/labstreaminglayer/App-AudioCapture), captured at 44100 Hz through WASAPI (Windows Audio Session API). Figure 4 illustrates the summary of the data acquisition system used in this study, including the network and the software depictions.

To further clarify and expand on potential uses of these recordings, we give an example where the audio data was analyzed to investigate filler duration in the subjects’ responses. Specifically, the dialogue data from our Driving Simulator study, consisting of questions by the system and answers by the participants, contain a lot of pauses and /uh/ and /um/ tokens, especially at the initiation of the responder’s vocalization. We analyzed the relation between the nature of the filler tokens and the subsequent delay and found that the duration of fillers such as /uh/ and /um/ is predictive of subsequent speech delays, and we were able to model these delays with high accuracy using the audio data. For example, we found that

1 $$d={f}_{{\rm{dur}}}\times f$$

where *d*, *f*_dur_, and *f* represent the delay, the filler duration, and the factor, respectively. In that study, we found out that the factor associated with /uh/ is 0.73 and the factor associated with /um/ is 1.24. This result provided a quantitative basis for the function of speech fillers introduced by Clark *et al*.^10^. A similar approach could be employed to further analyze

- The association between speech planning and cognitive workload via the timing and type of fillers.
- Driver stress or hesitation through prosodic features (pitch, intensity, tempo)^11^.

The audio recordings (including both simulator output and participant speech) offer useful opportunities for future research. In addition to studying speech fillers like /uh/ and /um/, researchers can explore how changes in voice (such as pitch, speed, or pauses) relate to cognitive workload, stress, or hesitation. The recordings also make it possible to study how people plan and respond during conversation under pressure. Since the audio is synchronized with other signals like EEG and eye tracking, it can support deeper analysis of how speech relates to cognitive state in real time.

### Procedure

In this double-session study, each participant was provided with an informed consent document and verbally explained their rights as participants in accordance with the Declaration of Helsinki and Tufts Internal Review Board. Afterward, the participants completed surveys on driving history and demographics. Next, they were brought to the driving simulator and were set up with the physiological monitoring equipment. The experiment consisted of two driving scenarios: one with the DRT and one without the DRT. The order in which these scenarios were presented was counterbalanced among participants. The DRT was set up and introduced to half of the participants at this time; the other half of the participants were set up and introduced to the DRT during the break before the second half of the experiment. Participants were then introduced to the driving simulator. They were instructed to stay in the right lane for the entire drive and to maintain a comfortable and appropriate speed while keeping in mind the posted speed limit of 65 mph.

Each scenario was 52.4 km long and took approximately 20 min to complete. The beginning of the drive consisted of 5.4 km (approximately 3 min) of just driving to allow the driver to acclimate to the simulation. After this section of the drive, the DRT began in one of the two drives (counterbalanced across participants). For the remainder of the scenarios (regardless of the presence or absence of the DRT), participants periodically engaged in six braking events and in four lure braking events as well as multiple dialogue communications. There were three secondary events along with the primary driving which we listed as follows. At that poinst, we would like to clarify that the same recording setup was used across all three scenarios—braking, dialogue, and DRT for ensuring consistency in data collection. The scenarios differ only in the tasks performed by participants, not in the technical configuration or recording methodology.

#### DRT

A DRT cylindrical vibrotactile motor was attached to the participants’ right collar shoulder just above the collar bone, measuring 14 mm in diameter and 4.5 mm thick. A response button with hook and loop tape was attached to the participant’s right index fingertip. Participants were instructed to respond to tactile stimuli by pressing the response button, which occurred randomly every 6 s to 10 s. The duration of the vibration and response logging/filtering followed ISO standards where the motor vibrated for 1000 ms or until the button was pressed, whichever came first. All responses below 100 ms and above 2500 ms were marked as missed responses (ISO 17488, 2016).

#### Braking Events

Braking events consisted of a vehicle appearing 200 m in front of the driver. Participants approached this lead vehicle until it was 75 m ahead and then followed this lead vehicle at a fixed distance of 75 m for 20 s. At that point, the lead vehicle rapidly decelerated for 5 s while its brake lights activated. After a braking event, the lead vehicle rapidly accelerated away from the driver. Lure braking events were similar to real braking events; however, after 20 s, the lead vehicle accelerated away from the driver and did not brake. We included lure braking events to minimize the risk of participants being able to anticipate a real braking event. The braking and lure braking events were spaced out throughout the drive so that they were approximately 1 to 3 minutes apart. The events were presented in different orders across participants to minimize any possible impact of order effects. The scenarios were otherwise identical in terms of the number and type of events.

#### Dialogue Interactions

During each scenario, participants responded to a series of basic fact questions about themselves. Twenty questions were asked during each drive (for a total of forty different questions), occurring roughly every 30 s to 60 s. Some of them are “yes/no” questions, and some of them are explanation questions that require the participants to respond with higher cognitive effort than “yes/no” questions. Table 1 shows the scripts used in this study. Here, E and Y/N indicate “explanation’ and “yes/no”, respectively. In addition, participants were allowed to rest briefly between the two scenarios. After the second scenario, participants filled out a final questionnaire that asked about aspects of the drive and contained the “simulator sickness questionnaire” (SSQ). The participant completed the experiment and post-experimental surveys within approximately 120 min.

**Table 1:** The list of the dialogue interactions used in this study, including the dialogue id, the type of the dialogue, and the detailed script.

ID	Type	Script
1	E	hi, I am molly
2	Y/N	do you watch netflix
3	E	are you an introvert or extravert
4	Y/N	do you have a tattoo
5	E	where do you live
6	E	do you prefer cities or nature
7	Y/N	do you wear glasses
8	Y/N	do you drink tea
9	Y/N	do you have triple a
10	Y/N	can you still hear me
11	E	what is your middle name
12	E	what type of food do you like
13	Y/N	are you right handed
14	E	how often do you drive
15	Y/N	do you watch the news
16	E	thanks for coming in
17	Y/N	do you have an iphone
18	Y/N	do you like cooking
19	E	what is your favorite season
20	Y/N	did you eat breakfast
21	E	what pets do you have
22	E	are you a leftie or rightie
23	Y/N	do you have a nickname
24	Y/N	do you drink coffee
25	E	what type of movies do you like
26	E	what sports do you watch
27	Y/N	do you have siblings
28	Y/N	do you read books
29	E	how is the weather outside
30	E	what is your eye color
31	Y/N	are you a student
32	Y/N	do you bike to work
33	Y/N	am I too loud
34	Y/N	did you drive here
35	E	when is your birthday
36	Y/N	are you driving
37	E	how are you today
38	E	how old are you
39	E	what is your favorite color
40	E	where are you from

There were 10 braking events in total in one session meaning that each participant completed 20 braking events in total during the entire experiment. Similarly, each participant was asked 20 questions per session (40 questions in total during the experiment). In addition, there were 5 SOA events per session (10 SOA occurrences in total during the experiment). DRT happenings were included every 6-10 seconds in which the number of occurrences slightly differ participant-to-participant. The response time to any braking or DRT event does not affect the next event happening, the consecutive events were independent. We will make sure to include this information to the revised manuscript.

**Fig. 5:**
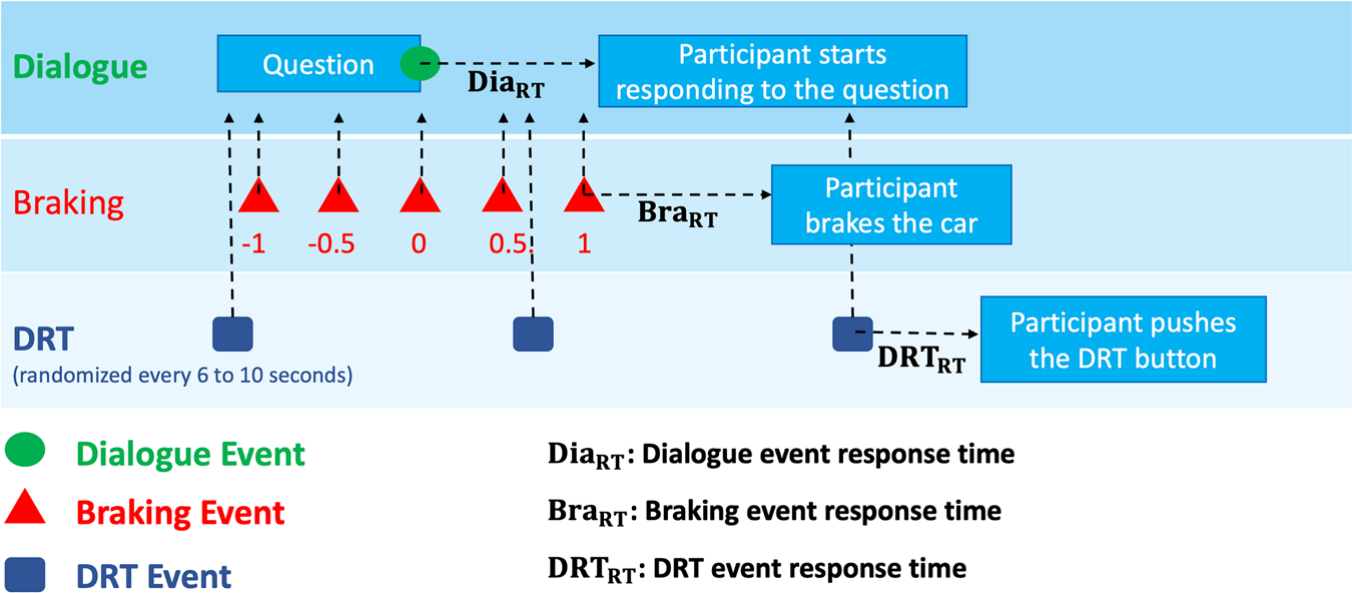
The schematic illustrating the relationship between communication, braking, and DRT events in the time domain (units in seconds).

To evaluate the effect of the braking occurrences on communication performance, and vice versa, we adjusted the relative timing of the braking events and dialogue interactions (see Fig. 5 showing the condition with DRT). The time difference between the onset of the braking event and the end of the question (script) was regulated from −1 to +1 seconds, with a step size of 0.5 seconds. We called this offset as SOA. A positive SOA value and a negative SOA value mean that the braking event occurs before the end of the presented question and the braking event occurs after the end of the question, respectively.

### Ethics approval

The approval number was “Tufts SBER IRB #1802002” and the approval date was “February 14, 2018”. The PI was Matthias Scheutz, and the Co-Investigators were JP de Ruiter, Shuchin Aeron, Nathan Ward, Sergio Fantini, Jason Rife. It was approved for 300 participants for the duration of the study.

Ethical approval to conduct the data collection for this study was obtained from the Tufts University Social, Behavioral and Educational Research IRB. The research protocol was reviewed in accordance with the requirements set forth by the Office for Human Research Protections (45 CFR 46) and the Food and Drug Administration (21 CFR 50).

The IRB reviewed the proposed protocol under the abbreviated Investigational Device Exemption (IDE) requirements as outlined in 21 CFR §812.2(b)(1) and determined that the study qualifies as a non-significant risk (NSR) device study. The IRB approval confirms that the study met all applicable ethical and regulatory standards for human subjects research.

### Consent to participate and data sharing

All participants in this study provided informed consent not only for their participation but also specifically for the open sharing of de-identified data. During the consent process, participants were clearly informed that:

- Their data would be de-identified to remove any personally identifying information.
- All audio and video recordings would be stored without identifying information and any potentially identifying features would be obscured prior to open sharing.
- Transcripts of dialogue tasks would be de-identified using pseudonyms.
- De-identified audiovisual recordings and transcripts would be made publicly available in an open-access online repository for research, teaching, and presentation at professional conferences.
- De-identified numeric and physiological data could be shared with other researchers for secondary analysis or reproduction of results.
- No data linked to participants’ contact information or identities would be shared with anyone outside of the Tufts research team.

Additionally, the consent procedure was reviewed and approved by the Social, Behavioral, and Educational Research IRB under Office of the Vice Provost for Research at Tufts University.

### Consent for publication

All authors have reviewed the final version of the manuscript and have given their consent for its publication. Participants gave explicit consent for these uses of their data, including the open-access sharing of de-identified materials. The participants also had a box to check: “I agree to be audio/video-taped for research and publication: YES NO Initial____”

## Data Records

**Fig. 6:**
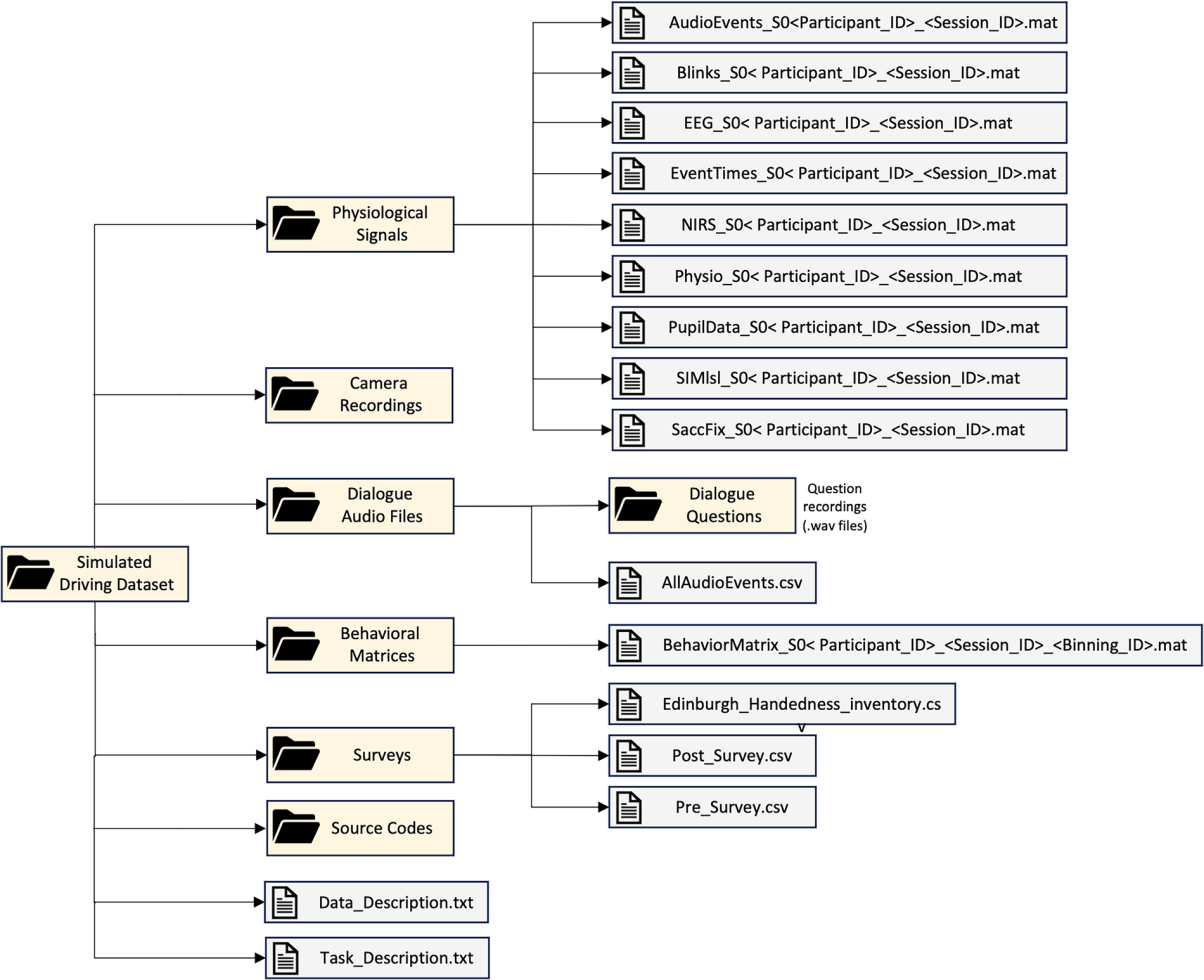
The taxonomy schematic represents the directory hierarchy of the simulated driving dataset.

The dataset, titled “Multi-modal Data Acquisition Platform for Behavioral Evaluation”, is available from^12^. Figure 6 shows the taxonomy representation of the directory hierarchy of the dataset. The parent directory involves four sub-directories, which are *Physiological Signals*, *D*ialogue Audio Files, *Behavioral Matrices*, and *Surveys*, in addition to two files called “Data_Description.txt” (detailed explanation of the data streams) and “Task_Description.txt” (experimental design specifications). Moreover, Table 2 shows the specifications of the signals, including the name of the data stream, the type of the signal, the description, and the sampling frequency of the signal.

**Table 2:** Summary of the packaged data streams including the signal type, the description of the data stream, and the sampling frequency.

Data Stream	Description	Sampling Frequency
EEG	**1-** Packet recordings from EEG electrodes.	
	**2-** 8 x n array of double-precision floating point values, where n is the number of packets recorded.	500 Hz
	**3-** Eight EEG channels acquired: FC5, FC1, CP5, CP1, CP2, CP6, FC2, and FC6.	
	**1-** Audio recording of the simulator output.	
AudioIn	**2-** 2 x n array of single-precision floating point values, where n is the sampled audio data.	44100 Hz
	**3-** This includes the questions from the dialog and simulator background noise.	
	**1-** Audio recording of the microphone attached to the front of the participant. This audio stream contains the answers to the dialogue events during the experiment.	
Mic	**2-** 2 x n array of single-precision floating point values, where n is the sampled audio data.	44100 Hz
	**3-** Only the first channel has meaningful data.	
	**1-** Raw optical data from the fNIRS sensors.	
fNIRS	**2-** 81 x n array of single-precision floating point values, where n is the sampled data.	7.8125 Hz
	**1-** Data from the physio suite of sensors.	
Physio	**2-** 4 x n array of double-precision floating point values, where n is the number of samples.	20 Hz
	**3-** This data includes blood pressure (in mmHg), skin conductance (in muS, respiration readings (in z scores), and blood *O*_2_ (in percentage).	
Pupillometry	**1-** Data from the PupilLabs eye tracker.	
	**2-** 22 x n array of double precision floating point values.	400 Hz
SIM	**1-** Data from the simulation software.	
	**2-** 1 x n array of double-precision floating point values, where n is the number of samples.	60 Hz
	**3-** The data vector corresponds to the simulator’s timestamps. Simulator data was acquired externally proper to the LSL protocol but is related to the LSL timestamp via the simulator’s timestamp.

1. **Physiological Signals:** This folder includes nine types of “.mat” files. Here, “Participant_ID” and “Session_ID” represent the participant number and the trial number, respectively.
  - **Audio Events:** “AudioEvents_S0 < Participant_ID > _ < Session_ID > .mat” files include 9 × *n* cell arrays where *n* is the number of dialogue events that occurred during the trial. There are usually 20 dialogue happenings within each trial, although there are a few trials that contain less than 20 dialogue events. The columns of the AudioEvents arrays include the ID of the participant, the ID of the session (either 1 or 2), the audio track identifier (a number between 1–40), the text of the audio (the question), the starting time of the audio (in seconds since the onset of the trial), the ending time of the audio (in seconds since the onset of the trial), the SOA time representing the time gap between the end of the question and the illumination of the brake lights of the car in front of the participant. (in seconds), the reaction time measure representing the time delay between the end of the question and the participant’s answer (in seconds), and the duration of the participant’s answers (in seconds).
  - **Blinks:** “Blinks_S0 < Participant_ID > _ < Session_ID > .mat” includes 12 × *n* arrays where *n* represents the number of blinks. This array includes multiple blinking properties including the onset point, the ending point, and the duration of the blink.
  - **EEG Signals:** “EEG_S0 < Participant_ID > _ < Session_ID > .mat” files contain information about EEG recordings. The EEG data is initially band-pass filtered from 0.25 Hz to 125 Hz. The data is also common mode averaged, meaning that each EEG channel is referenced against the average of all of the channels to attenuate the movement artifacts. The EEG consists of 9*xn* arrays where *n* is the number of samples. The first row represents the LSL timestamp (in seconds), and the rest of the eight rows represent the EEG channels, which are FC5, FC1, CP5, CP1, CP2, CP6, FC2, and FC6 (in uV).
  - **Event Times:** “EventTimes_S0 < Participant_ID > _ < Session_ID > .mat” files involve information about braking and DRT events such as the DRT response time (the LSL time in seconds that the participant responded to the DRT stimulation), the DRT signals on (the LSL time in seconds that the DRT signal is active), the DRT response time (the reaction time in seconds from the DRT signals on to the participant’s response), the brake lights on (the LSL time in seconds when the forward car’s brake lights turn on), and the brake reaction time (the LSL time in seconds of when the brake angle changed from the time the brake light turned on). There are ten braking occurrences per session. Among these ten braking happenings, four of them are luring events, where the car is positioned in front of the driver but does not break.
  - **NIRS Signals:** “NIRS_S0 < Participant_ID > _ < Session_ID > .mat” files include raw fNIRS output which was stored in a 42 × *n* arrays where *n* is the number of samples. The first row, the second row, and the next 40 rows represent the LSL timestamp (in seconds), the frame number, and the row fNIRS data, respectively.
  - **Physio Signals:** Inside “Physio_S0 < Participant_ID > _ < Session_ID > .mat” files, there are 5 × *n* arrays where *n* is the number of samples. The first, second, third, fourth, and fifth rows represent the LSL time (in seconds), the blood pressure signal (in mmHg), the skin conductance signal (in muS), the respiration signal (in z scores), and the *O*_2_ signal (in percentage), respectively.
  - **Pupillometry Signals:** “PupilData_S0 < Participant_ID > _ < Session_ID > .mat” files contain two 3 × *n* arrays representing the 2D model and the 3D model of the pupillometry signals where *n* is the number of samples. Each array includes three rows: the LSL timestamp, the left pupillometry signal, and the right pupillometry signal.
  - **Simulation:** “SIMlsl_S0 < Participant_ID > _ < Session_ID > .mat” files contain 31 × *n* arrays where *n* represents the number of samples. The first row indicates the LSL timestamp. Some of the rows involved in the simulation array are the degree of the brake angle (in degree), the braking time (in seconds), the DRT onset time (in seconds), and the brake force (in N).
  - **Saccades and Fixations:** “SaccFix_S0 < Participant_ID > _ < Session_ID > .mat” files include the information regarding saccadic movements and fixations. Specifically, the starting and the ending times of the saccades and the fixations (in seconds), as well as the duration of the saccades and the fixations (in seconds), are included in these files.
2. **Camera Recordings:** We stored world camera video recordings in this folder. There are two recordings per participant called “world_s1.mp4” and “world_s2.mp4” which represent the video file associated with the first session and the video file associated with the second session, respectively.
3. **Dialogue Audio Files:** In the “Dialogue Audio Files” folder, there is one file named “Dialogue Questions” in which there are audio recordings (.wav) corresponding to 40 dialogue questions. In addition to audio files, there is a document named “AllAudioEvents.csv”, which includes the ID of the participant, the session ID, the question ID (between 1–40), the script of the question, the starting LSL time of the question, the ending LSL time of the question, and the SOA value. Specifically, we adjusted the time gap between the onset of the braking event and the ending time of the question, *i.e*., the SOA, between −1 s and +1 s with a step size of 0.5 s. SOA values of −1 s and −0.5 s represent the cases where the braking event happens 1 s and 0.5 s after completing the question, respectively. Similarly, SOA values of 1 s and 0.5 s represent the cases where the braking event occurs 1 s and 0.5 s before completing the question, respectively. Moreover, SOA of 0 indicates that the question ends at the same time as the braking event begins. The SOA values in the document approximate 1 s, 0.5 s, −0.5 s, or −1 s, though they might deviate slightly. Moreover, NaN values represent the dialogue communications that are not involved in an SOA happening.
4. **Behavioral Matrices:** During the experiment, we identified 13 logical markers for specific time intervals. The file name is “BehaviorMatrix_S0 < Participant_ID > _ < Session_ID > _ < Binning_ID > .mat”. The Binning_ID ranges from 1 through 10 (in seconds), which determines the size of the behavior matrix array. For example, “BehaviorMatrix_S0101_1_1.mat” is a 1450 × 13 logical array (we assign a logical marker to each 1 s timeframe), besides “BehaviorMatrix_S0101_1_10.mat” is a 145 × 13 logical array (we assign a logical marker to each 10 s timeframe). The logical markers include missed DRT signal, slow DRT response, fast DRT response, slow communication response, fast communication response, slow braking reaction, fast braking reaction, slow steering, fast steering, low position offset, high position offset, low heading offset, and high heading offset. The definitions related to 13 logical markers can be found in “Data_Description.txt”.
5. **Surveys:** In the Surveys folder, there are three documents. In “Edinburgh_Handedness_Inventory.csv”, there are questions including participants’ preferences on using their hands and feet for specific tasks such as writing, toothbrush, scissors, throwing, or using a spoon. “Post_Survey.csv” includes questions about participants’ physical or mental conditions after the experiment, such as feeling stressed, having difficulty focusing and concentrating, sweating, dizziness with eyes open, or having headaches. Finally, “Pre_Survey.csv” contains general questions such as the participants’ age, their current physical well-being, their current mental well-being, whether they use corrective lenses or not, the rate of their overall hearing, or how often they drive a car or other motor vehicles.

## Data Overview

The self-report surveys administered included measures of participants’ subjective states before and after the experimental tasks, including fatigue, stress, and a range of physical and cognitive discomfort symptoms. These surveys were intended to contextualize the physiological and behavioral data, providing insight into participants’ somatic and observational responses to the experimental setup.

Here we include a brief summary of the post experimental survey results:

- **Fatigue and Drowsiness:** Drowsiness levels ranged widely (1–10), with a cluster around 6–8 indicating moderate tiredness. While some participants reported feeling quite alert, others reported high levels of fatigue, which may be linked to the length or cognitive demand of the tasks.
- **Stress:** Stress levels also varied, though most participants rated their stress as moderate (3–5). A smaller subset reported high stress (7–9), often correlating with more intense physical symptoms or discomfort from the equipment.
- **Discomfort Symptoms:** Across the group, the most commonly reported symptoms were headache, moderate dizziness and nausea, and finger discomfort and numbness.

Several participants noted that despite discomfort, they found the study engaging or enjoyable. However, a subset reported that physical symptoms such as headaches, nausea, or sensory pressure were significant enough to impair concentration or create a desire to withdraw early.

These survey responses are critical for interpreting variations in physiological signals and task performance. For example, physical discomfort, particularly headaches and dizziness, may affect attention levels and could correspond with behavioral markers of reduced task engagement. Participant feedback also helps identifying potential confounds in data interpretation, particularly where physical discomfort (e.g., from equipment) may mimic or mask cognitive fatigue or stress responses.

## Technical Validation

**Fig. 7:**
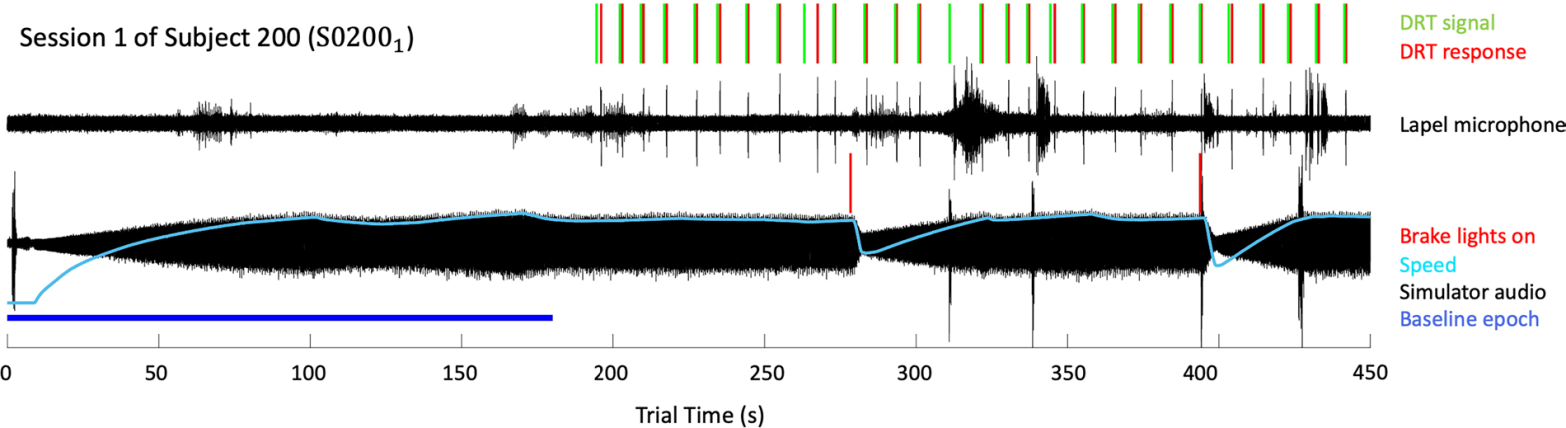
The illustration of the timing between the data streams.

Figure 7 shows synchronized data from the three recorded signal streams and two audio channels (as described in^12^). Alignment of vehicle speed, braking events, dialog playback, and participant responses confirms accurate temporal synchronization. The lapel mic also captured incidental DRT clicks, which align with DRT events recorded by the simulator, providing further cross-validation. These checks support the dataset’s accuracy and reliability for reuse.

## Data Availability

The complete dataset entitled “Multi-modal Data Acquisition Platform for Behavioral Evaluation” is available from^12^.

## Code availability

In this study, we used Matlab to import and process the raw data. We provided the code which we used to acquire and process the data under the sub-folder named “Source Code” (refer to Figure 6 for data taxonomy and see the repository in^12^).
